# Down-Selection and Outdoor Evaluation of Novel, Halotolerant Algal Strains for Winter Cultivation

**DOI:** 10.3389/fpls.2018.01513

**Published:** 2018-10-29

**Authors:** Lukas R. Dahlin, Stefanie Van Wychen, Henri G. Gerken, John McGowen, Philip T. Pienkos, Matthew C. Posewitz, Michael T. Guarnieri

**Affiliations:** ^1^Department of Chemistry, Colorado School of Mines, Golden, CO, United States; ^2^National Renewable Energy Laboratory, National Bioenergy Center, Golden, CO, United States; ^3^Arizona Center for Algae Technology and Innovation, Arizona State University, Mesa, AZ, United States

**Keywords:** microalgae, algal biofuels, algae screening, algae composition, winter algae, outdoor winter cultivation

## Abstract

Algae offer promising feedstocks for the production of renewable fuel and chemical intermediates. However, poor outdoor winter cultivation capacity currently limits deployment potential. In this study, 300 distinct algal strains were screened in saline medium to determine their cultivation suitability during winter conditions in Mesa, Arizona. Three strains, from the genera *Micractinium, Chlorella*, and *Scenedesmus*, were chosen following laboratory evaluations and grown outdoors in 1000 L raceway ponds during the winter. Strains were down-selected based on doubling time, lipid and carbohydrate amount, final biomass accumulation capacity, cell size and phylogenetic diversity. Algal biomass productivity and compositional analysis for lipids and carbohydrates show successful outdoor deployment and cultivation under winter conditions for these strains. Outdoor harvest-yield biomass productivities ranged from 2.9 to 4.0 g/m^2^/day over an 18 days winter cultivation trial, with maximum productivities ranging from 4.0 to 6.5 g/m^2^/day, the highest productivities reported to date for algal winter strains grown in saline media in open raceway ponds. Peak fatty acid levels ranged from 9 to 26% percent of biomass, and peak carbohydrate levels ranged from 13 to 34% depending on the strain. Changes in the lipid and carbohydrate profile throughout outdoor growth are reported. This study demonstrates that algal strain screening under simulated outdoor environmental conditions in the laboratory enables identification of strains with robust biomass productivity and biofuel precursor composition. The strains isolated here represent promising winter deployment candidates for seasonal algal biomass production when using crop rotation strategies.

## Introduction

Algae are a promising source of renewable biomass that can be converted to biofuels and bioproducts. Of the various terrestrial feedstocks currently grown at large scales, algae have the potential to achieve orders of magnitude higher productivities and can be grown on land not currently suitable for food production (Chisti, 2007). Algae can also mitigate the problems associated with climate change by reducing the need to burn additional fossil fuels. In contrast to fossil fuels, algae utilize CO_2_ already in the atmosphere and can potentially be integrated with power plants to consume CO_2_ emissions from the generation of electricity or other point source CO_2_ emissions. Furthermore, many algae are able to use saline or brackish water resources which should reduce costs and avoid further demands on limited freshwater resources. Lastly, algae can be grown throughout the calendar year in many locations. However, despite the apparent promise of algal biomass, algae have yet to be fully utilized at industrial scales, due in part to the poor correlation of productivities measured in the laboratory to outdoor performance and ultimately lack of cost competitiveness with traditional petroleum-based fuel resources at current prices (Sheehan et al., 1978; Quinn and Davis, 2015).

To realize the potential of algae, significant productivity improvements are required (Quinn and Davis, 2015; Davis et al., 2016). Cost and sustainability improvements can be made through the use of halotolerant algae grown in saltwater. This is due to the global abundance of saltwater relative to limited freshwater resources, which is a critical consideration for contemporary agriculture and sustainability. Further improvements can be made if a seasonal crop rotation strategy is employed. There is typically a substantial decrease in algal productivities during the winter months at most locations, and the identification and use of superior winter strains will improve overall annual biomass yields if strains better suited to outdoor growth in colder conditions are deployed during this season (Sheehan et al., 1978; Quinn et al., 2012; Quinn and Davis, 2015; White and Ryan, 2015; Davis et al., 2016; Lammers et al., 2017). Unfortunately, it is often difficult to identify strains in controlled laboratory environments that can be successfully cultured outdoors. As noted by Griffiths and Harrison (2009), outdoor productivities are often diminished relative to laboratory yields due to the more extreme dynamics and levels of outdoor temperatures and light intensities, environmental conditions that are often not replicated during strain selection indoors. Therefore, we designed a growth system that better reflected the outdoor environmental parameters of light and temperature and then screened uncharacterized algal strains for isolates that perform well under real-world fluctuating temperature and light to identify strains with improved biomass productivities relative to previously tested isolates.

Herein, we targeted identification of halotolerant algal strains suitable for outdoor cultivation during winter for the production of biofuels and bioproducts. To achieve this, a previously uncharacterized algal culture collection was screened under simulated outdoor winter conditions using a seawater-based medium. As described by Elliott et al. (2012), this collection comprising novel strains was established via the isolation of algae from diverse water samples through the southwestern United States. Utilizing a custom-built photobioreactor, we screened this algal collection using real-world winter pond temperature and photosynthetically active radiation (PAR) inputs derived from 1000 L outdoor algal ponds located in Mesa, Arizona. After screening in simulated outdoor winter conditions, three diverse, high-potential, down-selected strains were deployed outdoors to verify and quantify their suitability for large scale biomass and biofuel production.

## Materials and Methods

### Culture Collection Screening

Preliminary screening efforts utilized a custom-built photobioreactor capable of simulating the variations in PAR and temperature common in outdoor raceway ponds. This was achieved using a 73.4 L polycarbonate aquarium (109.9 × 29.2 × 29.2 cm), connected to a programmable water chiller/heater circulating pump (VWR, Model AP15R-30-V11B). White LED (light emitting diode) lights (Lithonia, Model IBH24000LMSD080MDMVOLTGZ1040K70CRICS93WWH) illuminated both of the longer sides of the water bath, and were connected to a programmable dimmer (Pulsar, Model P02C1-375HS_SERIAL), this provided uniform lighting to all culture tubes. Carbon dioxide was mixed with air to 2% CO_2_ and delivered via rotameters to 36 individual tubes at 100 mL/min, which also provided aeration-mediated mixing to the 100 mL cultures. A schematic of this PBR is available in the Supplementary Material. Winter water temperature cycled from 6 to 14°C in a sinusoidal fashion daily which represents the average temperatures measured in 1000 L elevated raceway ponds at the Arizona Center for Algae Technology and Innovation (AzCATI) testbed site located at Arizona State University in Mesa Arizona from December 17th 2014 to January 28th 2015. At the same time, PAR cycled from 0 to 965 μmol m^−2^ s^−1^, over 9 h, followed by 15 h of darkness; this closely represented the PAR conditions at outdoors ponds at the time and location described above.

To initiate the screening process, 300 strains from diverse environments were first evaluated for their ability to grow in seawater-based media (defined below). Of the 300, 107 strains were found to be halotolerant (growth in seawater-based media) and were further screened in duplicate under simulated winter conditions. In each screening run, approximately 35 unique strains were each grown in single chambers, from a starting optical density (750 nm) of 0.2. To compare the most promising isolates, the top performing strains from each winter run were run for a 3rd time to produce triplicate data. This represented 33 promising winter isolates chosen by the down-selection criteria that included: doubling time, lipid and carbohydrate amount, final biomass accumulation capacity, cell size and phylogenetic diversity. During all runs, optical density (750 nm) was measured over a time course (measurements every other day), from which doubling time could be calculated, followed by endpoint compositional analysis, which included analysis of moisture, ash, lipid content as converted fatty-acid-methyl-esters (FAME), and carbohydrates. The reported doubling time, calculated during linear growth phase, includes time in which the lights were off simulating night time (15 h a day), and thus is a representation of a real-world winter doubling time including light and temperature variation rather than the shortest doubling time possible under ideal conditions. The cell size reported was calculated by averaging 20 linear phase cells measured with a Nikon Eclipse Ci microscope, utilizing NID Elements v. 4.40.00 software and calibrated with an improved Neubauer hemocytometer; average and the respective standard deviation is reported in the results.

The media used in screening was a modified f/2 formulation (Guillard and Ryther, 1962). To seawater (Gulf of Maine, Bigelow Laboratory) the following were added to the indicated final concentration followed by addition of 12 M HCl to attain pH 8.0: NH_4_Cl (5.0 × 10^−3^ M), NaH_2_PO_4_⋅H_2_O (0.313 × 10^−3^ M), Na_2_SiO_3_⋅9H2O (1.06 × 10^−4^ M), FeCl_3_⋅6H_2_O (1.17 × 10^−5^ M), Na_2_EDTA⋅2H_2_O (1.17 × 10^−5^ M), CuSO_4_⋅5H_2_O (3.93 × 10^−8^ M), Na_2_MoO_4_⋅2H_2_O (2.60 × 10^−8^ M), ZnSO_4_⋅7H_2_O (7.65 × 10^−8^ M), CoCl_2_⋅6H_2_O (4.20 × 10^−8^ M), MnCl_2_⋅4H_2_O, (9.10 × 10^−7^ M), thiamine HCl (2.96 × 10^−7^ M), biotin (2.05 × 10^−9^ M), cyanocobalamin (3.69 × 10^−10^ M), Tris base (24.76 × 10^−3^ M).

### 18s Sequencing

Phylogenetic sequencing was conducted using universal eukaryotic primers to amplify the 18S rRNA gene. Reverse primer, 1391RE 5-GGGCGGTGTGTACAARGRG-3 and forward primer 360FE 5-CGGAGARGGMGCMTGAGA-3 were used for all amplifications (Dawson and Pace, 2002). Colony PCR was conducted wherein a single colony was picked from a solid agar matrix, placed in 20 μL of Y-PER lysis buffer (Sigma), boiled for 5 min, and diluted by addition of 150 μL of H_2_O. PCR was carried out with 2 μL of the cell lysate, 25 μL of Q5^®^ Hot Start High-Fidelity 2X Master Mix (New England Biolabs), 18 μL of H_2_O, and 2.5 μL each of the forward and reverse primers (10 μM) (Packeiser et al., 2013). The thermocycler protocol is as follows: 94°C initial denature for 30 s, followed by 30 cycles of 94°C denaturation for 30 s, 63°C annealing for 15 s, 72°C elongation for 1 min and 20 s, a 2 min final elongation at 72°C was utilized. The 18S amplicon was sequenced (Genewiz) and the NCBI BLAST algorithm (NCBI Resource Coordinators, 2017) used as an initial determination of speciation of the novel strains. The above methods were also used to verify the speciation in outdoor growth, DNA was extracted using a MasterPure^TM^ Yeast DNA Purification kit (Lucigen) from day 0 seed cultures, lyophilized biomass samples, ultimately confirming correct speciation via 18s sequences. 18s sequences were deposited into the Sequence Read Archive, submission ID: SRP136934.

### Outdoor Growth

Following down-selection via indoor screening, 3 promising winter isolates 14-F2 (*Micractinium reisseri*), 4-C12 (*Chlorella vulgaris*), and 46B-D3 (*Scenedesmus rubescens*) were deployed outdoors utilizing the AzCATI testbed site located at Arizona State University in Mesa Arizona during February of 2016. Cultures were scaled up indoors using bubble columns and 15 L flat panel reactors as described in McGowen et al. (2017), and finally inoculated into 1000 L raceway ponds at a 25 cm depth, with an area of 4.2 m^2^. Media consisted of 35 g/L salinity achieved using Crystal Sea (Marine Enterprises International), 2.14 mM ammonium chloride and 0.134 mM phosphate (NaH_2_PO_4_⋅H_2_O). Trace metals were added to the following final concentrations (standard f/2 media), FeCl_3_⋅6H_2_O (1.17 × 10^−5^ M), Na_2_EDTA⋅2H_2_O (1.17 × 10^−5^ M), CuSO_4_⋅5H_2_O (3.93 × 10^−8^ M), Na_2_MoO_4_⋅2H_2_O (2.60 × 10^−8^ M), ZnSO_4_⋅7H_2_O (7.65 × 10^−8^ M), CoCl_2_⋅6H_2_O (4.20 × 10^−8^ M), MnCl_2_⋅4H_2_O, (9.10 × 10^−7^ M). Inoculation density was targeted at 0.05 g/L of algal biomass, and sodium carbonate was added as necessary to control the pH drop due to consumption of NH_3_ from the added NH_4_Cl. Pond pH was adjusted to 7.9 via a pH controller connected to a solenoid delivering CO_2_ gas, and PAR was measured continuously with a LiCor LI-190R quantum pyranometer (McGowen et al., 2017). Freshwater was added prior to sampling to account for evaporative loses. Biomass samples for analysis were taken in the morning (07:30–08:00), except for inoculation biomass which was sampled at (15:00). Qualitative microscopic examination of outdoor cultures was conducted periodically to assess contamination. In order to compare strains, two productivity metrics were used. The first was productivity calculated during linear phase growth, termed maximum productivity here. The second, harvest-yield productivity, is a measure of the net productivity over the course of the entire cultivation trial, which includes the lag, linear, and stationary culturing phases. The end of the harvest-yield was chosen when cultures were not adding significant biomass during the day, and nutrients had not been measurable for multiple days in the media.

### Ash Free Dry Weight, Fatty-Acid-Methyl-Ester, and Carbohydrate Analysis

Compositional analysis for ash (Van Wychen and Laurens, 2013b), FAME (Laurens et al., 2012; Van Wychen and Laurens, 2013a), and carbohydrate (Templeton et al., 2012; Van Wychen and Laurens, 2013c) were conducted as previously reported. Briefly, residual moisture after lyophilization was determined by heating of pre-weighed algal biomass at 40°C for at least 18 h, followed by ash analysis by ramping at 20°C/min to 575°C, and held for 180 min. FAME analysis was conducted by transesterification of 10 mg of lyophilized algal biomass in 200 μL chloroform:methanol (2:1 v/v) and 300 μL 0.6M HCl:methanol (2.1% v/v) at 85°C for 1 h. FAMEs were extracted with 1.0 mL hexane and analyzed via a gas chromatograph with flame ionization detection (Agilent 7890A equipped with a split/splitless inlet and an Agilent J&W GC Column DB-Wax 30 m × 0.25 mm × 0.25 μm). Tridecanoic acid methyl ester (C13:0ME) was used as an internal standard. Carbohydrates were determined by hydrolysis of 25 mg lyophilized biomass with 250 μL of 72% (w/w) sulfuric acid at 30°C for 1 h, followed by dilution with 7 mL of water and autoclaving for 1 h at 121°C. Samples were filtered through a 0.2 μm nylon filter, and subjected to high pressure anion exchange liquid chromatography with pulsed amperometric detection on a PA-20 column (Thermo Scientific – Dionex ICS-5000 + Analytical HPIC System). For outdoor cultivation, ash free dry weight (AFDW) was tracked throughout outdoor growth using the methodology described in McGowen et al. (2017), which involved filtering the algal culture through a pre-weighed glass microfiber filter followed by drying, ashing and re-weighing. Three independent trials (biological triplicates) were conducted for all analyses; average and standard deviations were obtained for replicates.

## Results

### Indoor Screening

Initial down-selection involved screening for the ability of 300 strains to grow in saline media, as the algae were collected from diverse water sources with varied salinity. Of these 300, 107 showed growth in saline media, and were thus subjected to more detailed growth analysis as described in the methods. All data generated from this screening is available in the Supplementary Material, of the 107 halotolerant strains screened only 69 grew under the simulated winter conditions. Supplementary Table S1 shows the primary metrics used to determine further down-selection of the 107 strains, including biomass accumulation, FAME and carbohydrate content, and doubling time. Of the strains deployed outdoors, strain 14-F2 showed the highest net biomass accumulation (2.32 g/L) and shortest doubling time (∼40 h, including the dark period). Strain 4-C12 showed the highest FAME percentage (∼37%). Strain 46B-D3 had both the largest cell size (∼12 μm) and carbohydrate percentage (∼30%), along with a relatively rapid auto settling capacity and carotenogenesis (data not shown). When choosing which strains to deploy, outdoor biomass productivity and composition were used as primary selection criteria, however, qualitative observations of cell size, morphology, and auto settling capacity were also utilized to ensure a diverse set of organisms were cultivated outdoors. The diversity is ultimately reflected in the genus level speciation of the strains grown outdoors, 14-F2 (*Micractinium reisseri*), 4-C12 (*Chlorella vulgaris*), 46B-D3 (*Scenedesmus rubescens*); along with their respective cell sizes, 4.2 ± 0.5, 5.7 ± 0.7, and 11.7 ± 1.6 μm.

We attempted to use *Nannochloropsis salina* (CCMP 1776) as a baseline control in all down-selection trials. *Nannochloropsis* sp. are widely regarded as industrially relevant for outdoor growth due to their relatively high productivities, high lipid content, and stability during outdoor cultivation (Guillard and Ryther, 1962; Griffiths and Harrison, 2009; Rodolfi et al., 2009; Bondioli et al., 2012; Quinn et al., 2012; Neofotis et al., 2016; Lammers et al., 2017). Furthermore, the United States Department of Energy recently defined the winter strain state of technology based upon *Nannochloropsis oceanica* (KA32) productivity (Office of Energy Efficiency Renewable Energy, 2017). Notably, *Nannochloropsis salina* and *Nannochloropsis oceanica* failed to grow under the simulated winter growth conditions employed here, likely due to the extreme cold temperatures employed. This observation further underscores the need for improved winter cultivation strains.

### Outdoor Deployment

Due to the highly controlled nature of indoor laboratory screening, which shields algae from many outdoor environmental stresses, the top winter strains were grown outside in Mesa Arizona at the AzCATI testbed site to determine whether the down-selected strains can be successfully cultivated outdoors during the winter growing season. Briefly, strains were cultivated in 1000 L raceway ponds during February 2016, which led to a cold start to cultivation as discussed below, providing a well-suited winter experiment, which tested the extreme cold tolerance of the strains.

**FIGURE 1:**
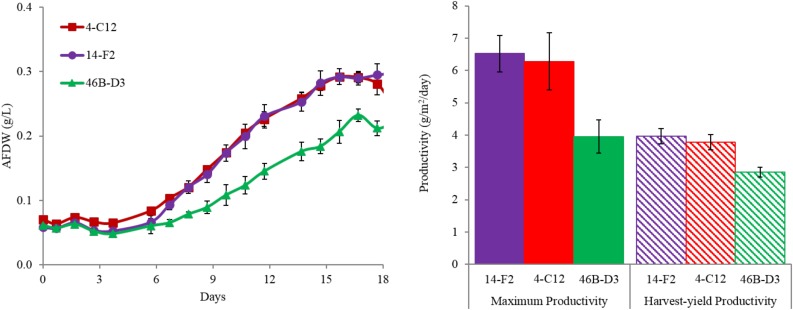
(Left) Ash-Free Dry Weight accumulation and (right) areal productivities for strains cultivated outdoors during the winter month of February 2016 (1st–19th). Maximum productivity is calculated from the steepest portion of growth curve, while harvest-yield productivity measures all biomass accumulated up to day 18. Average and standard deviation error bars are from 3 biological replicates.

**FIGURE 2:**
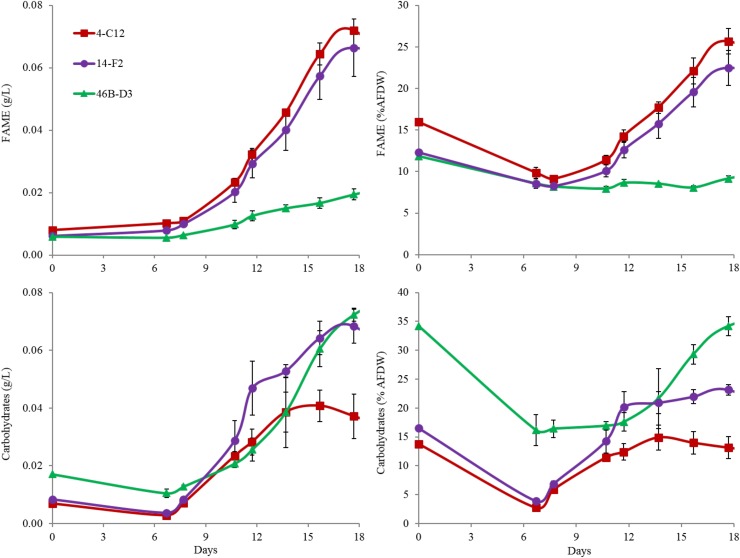
FAME and Carbohydrate analysis of strains grown outdoors during the winter month of February 2016 (1st–19th). Average and standard deviation error bars are from 3 biological replicates.

**FIGURE 3:**
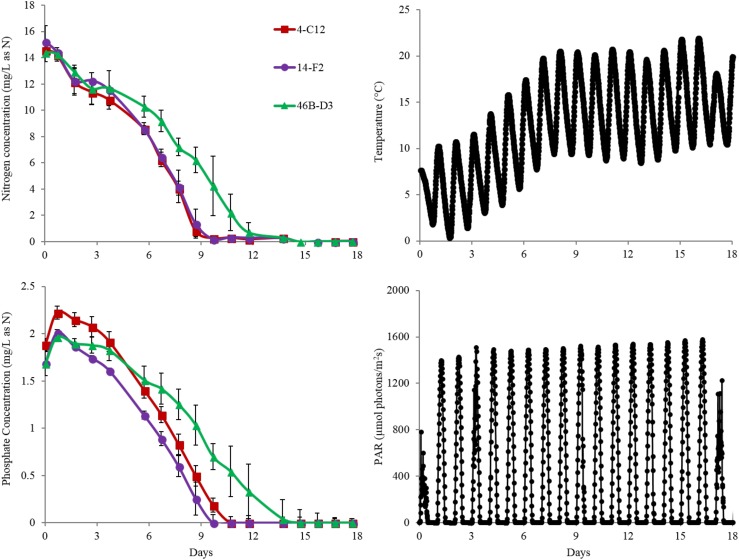
Nitrogen (as ammonium) depletion of strains grown outdoors during the winter month of February 2016 (1st–19th) (Top Left). Phosphate depletion of strains grown outdoors under winter conditions (Bottom Left). Representative change in temperature of ponds throughout the experiment (Top Right). Representative change in PAR of ponds throughout the experiment (Bottom Right). Average and standard deviation error bars are from 3 biological replicates.

Overall harvest-yield biomass productivities were greatest for both 14-F2 and 4-C12, with productivities of 4.0 and 3.8 g/m^2^/day, respectively. Strain 46B-D3 showed the lowest productivity of 2.9 g/m^2^/day (Figure 1). Maximum productivities showed a similar trend, however were naturally greater, as this excluded the ∼6 days long lag phase (Figure 1). It is interesting to note that indoor biomass titers were as high as 2.32 g/L, whereas outdoor titers only reached 0.295 g/L (Supplementary Table S1 and Supplementary Figure S1). This represents approximately an order of magnitude change in biomass titers from indoor to outdoor cultivation, underscoring the need to assess strains under real world outdoor conditions. Qualitative microscopic examination of outdoor cultures indicated negligible amounts of contaminating organisms in comparison to the alga of interest. It is important to note that the strains were grown in relatively cold temperatures, mostly varying between 10 and 20°C, with a cold minimum (0°C) early in cultivation (Figure 3). Additionally, the lower winter light duration and intensities tend to decrease growth rates and lipid content (Khotimchenko and Yakovleva, 2005). Actual light intensities measured during the experiment are shown in Figure 3. PAR peaked at approximately 1475 μmol photons/m^2^s, with measurable light for ∼10.5 h each day and 13.5 h of darkness. Ammonium and phosphate consumption correlated with algal growth. Ammonium concentration in the media was not detectable at day 9 for strains 4-C12 and 14-F2, and at day 12 for 46B-D3, with phosphate concentrations showing a similar trend (Figure 3). Significant biomass accumulation was observed after nutrients were depleted, indicating active carbon assimilation and photosynthesis (Figure 1). As seen in Figure 2, the percentages of FAMEs and carbohydrates begins to increase at the approximate time that nutrients are depleted in the media, however, carbon fixation continues after this as AFDW continues to increase. Putative chlorophyll degradation was observed for all strains grown outdoors, as evident by a decrease in 680 nm/750 nm absorption ratios (data not shown).

**FIGURE 4:**
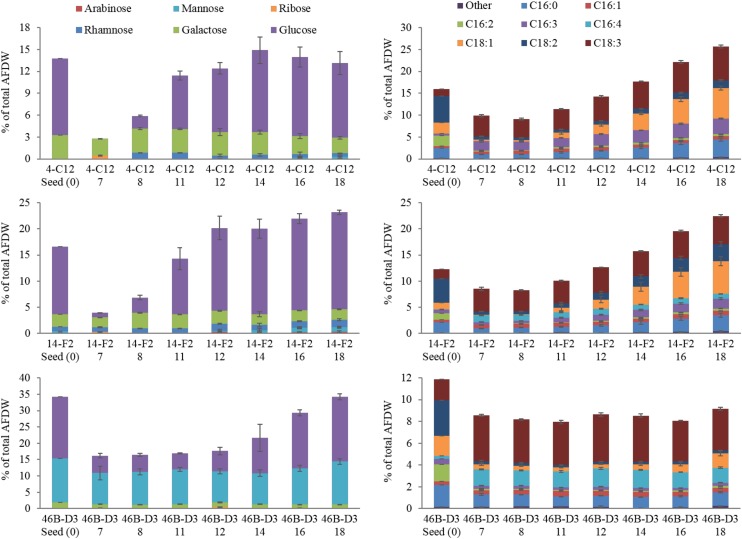
Carbohydrate and FAME profile of strains grown outdoors during the winter month of February 2016 (1st–19th), x axis lists the strain and time in days. Average and standard deviation error bars are from 3 biological replicates.

Perhaps most notable, considering the growth conditions, is the demonstration of a relatively high lipid state in two of the strains grown outdoors, 14-F2 and 4-C12, which showed peak FAME percentages of 22.5 and 25.8, and titers of 0.066 and 0.072 g/L, respectively (Figure 2). As shown in Figure 4, lipids contained almost exclusively 16 and 18 long carbon chains with varying degrees of unsaturation depending on the strain and time point. The change in fatty acid chain lengths for all strains was quite dramatic from inoculation to endpoint harvest, with a substantial decrease in 16:2 and 18:2 chain lengths after inoculation. Concurrently, a decrease in their respective saturation was observed. The increase in lipids for strains 14-F2 and 4-C12 was primarily due to accumulation of 18:1, 18:2, and 16:0 fatty acids, as shown in Figure 4. Strain 46B-D3 did not display an increase in lipid accumulation under the conditions tested.

Total carbohydrate accumulation is shown in Figure 2, which demonstrates that the down-selected strains are capable of accumulating high levels of total carbohydrates during winter months. Strains 14-F2 and 46B-D3 have the highest carbohydrate titers at ∼0.07 g/L at day 18, with 46B-D3 having the highest carbohydrate percentage at ∼34%. Figure 4 shows the sugar monomer content of hydrolyzed biomass. Glucose is heavily represented, however, measurable amounts of galactose, rhamnose, ribose and mannose are also present depending on the strain and time point. Notably, ∼40–60% of the carbohydrate fraction or 13% of the biomass in 46B-D3 is mannose. The most dramatic changes in sugar monomer profiles for all strains were observed at 7 days after inoculation into the outdoor raceway ponds when the strains were beginning to exit from their initial lag phase and entering maximum productivity. After this time, the carbohydrate profiles of each of the strains trended back toward the initial inoculation profile. All strains showed depletion in the amount of glucose from days 0 to 7. At day 7, strains 14-F2 and 4-C12 were largely composed of galactose in regard to sugar monomers; at the same time strain 46B-D3 was composed largely of mannose and glucose in regard to sugar monomers (Figure 4).

Another important observation to note is the relatively high FAME and carbohydrate percentages of the seed culture used to inoculate the raceway ponds, likely representing an early stationary phase culture (Figure 2). This is putatively due to the seed cultures being grown in flat panel photobioreactors with a decreased light path, allowing greater light saturation, and in turn, accumulation of energy storage molecules. Figure 2 clearly shows these energy storage molecules are utilized for cell growth and maintenance upon inoculation into the ponds with fresh media; as the percentages of both FAME and carbohydrates decrease from the time of inoculation to day 7. In fact, total carbohydrates (g/L) decreased for all strains from day 0 to 7. The cold temperatures the cultures experienced from days 0 to 6, likely required remobilization and utilization of stored carbon to survive (Figure 3).

## Discussion

### Indoor Screening

Both lipids and carbohydrates provide potential precursors for biofuels and bioproducts. Lipids can be directly converted to hydrocarbon-based fuels, whereas carbohydrates can be converted to fuel substitutes or valuable coproducts via fermentation in a CAP (combined algal processing) approach, and thus add additional value to algal biomass (Dong et al., 2015). It is clear that many algae can attain a high percentage of storage carbon, however, this metric alone does not necessarily predict overall lipid productivity on a volume or areal basis. Furthermore, it is well documented that outdoor growth in ponds does not necessarily correlate with the extremely high lipid storage state that is often observed in a laboratory setting or by the use of photobioreactors (PBRs) (Unkefer et al., 2016; Lammers et al., 2017). Therefore, both doubling time and overall biomass accumulation along with storage carbon percentage were used as selection criteria when deciding which strains would be deployed for outdoor testing.

Although the above criteria (as shown in Supplementary Table S1) were the most heavily factored attributes used in the down-selection process, other assets including organismal diversity, cell size, auto settling capacity, and the ability of strains to accumulate high value co-products, were also considered. With respect to diversity, it was hypothesized that moving forward exclusively with highly similar isolates could lead to common outdoor growth outcomes. For example, it is reported that many *Chlorella* isolates rapidly drop in productivity, ultimately requiring fresh inoculum in outdoor trials due to problems associated with predation and contaminating organisms, such as *Vampirovibrio chlorellavorus* (Coder and Starr, 1978; Ganuza et al., 2016). As such, genus level diversity was targeted in the final down-selection. Cell size and morphology is suggested to be a mechanism that algae use to avoid predation; therefore cells with unusually large cell size, or the tendency to clump or stack may be preferable for outdoor cultivation despite slightly lower productivities measured in the laboratory (Porter, 1973; National Alliance for Advanced Biofuels Bio-Products, 2014; White and Ryan, 2015). Algal morphological alterations have been observed in response to predation/predator infochemicals. Thus, sterile lab conditions may not correlate to the actual morphology observed during pond deployment, as previously reported for *Scenedesmaceae* and *Micractinium* (Luo et al., 2006; Yang et al., 2008; Wu et al., 2013), hence the logic in choosing these genera for the outdoor deployment reported here. The ability to avoid predation by this mechanism is simply conferred by the algal cell’s ability to become too large for many predator classes to consume. Another important metric is settling time, as this allows for greatly reduced volumes of water containing algae that need to be processed during dewatering. Gravity sedimentation is a relatively cheap primary harvesting technique that can concentrate algal biomass to 1.5% TSS (total suspended solids), from an initial TSS of ∼0.04% (Uduman et al., 2010). Deploying strains that inherently settle out of solution quickly could bypass the need for additional bio or chemical based flocculation, which contributes to the operational expenses of an algal farm, ultimately reducing final production costs (Brennan and Owende, 2010; Chen et al., 2011). The ability of strains to accumulate carotenoids or high value polyunsaturated fatty acids was also considered as these are generally considered high value co-products, which could be used to increase the cost competitiveness of algal biofuels (Brennan and Owende, 2010). A number of strains were noted to produce the high value omega 3 fatty acids, EPA (eicosapentaenoic acid) and DHA (docosahexaenoic acid); unfortunately, these strains displayed relatively low productivities under the tested conditions and were therefore not pursued past initial screening.

### Outdoor Deployment

The data presented here validated the indoor screening and selection methodology, with the down-selected strains showing growth and composition suitable for biofuel and bioproducts production when considering the cold temperatures of the ponds (Figures 1–3). These data represent the highest productivities reported to date for algal winter strains grown in saline media in open raceway ponds. This is in comparison to the productivities reported by McGowen et al. (2017), wherein similar cultivation conditions were utilized to grow *N. oceanica* (KA32). As discussed above, storage carbon accumulation and chlorophyll degradation correlate with nutrient depletion, however, carbon fixation continued, as reflected by AFDW increase. This suggests the cells are initiating a nutrient deprivation response once the nutrients in the media are depleted, despite the fact there are sequestered nutrients in the cells. This is supported by Li et al. (2008), wherein the authors postulated that nitrogen containing compounds such as chlorophyll are remobilized as a nitrogen source for cell growth once nitrogen in the media is depleted. These authors observed chlorophyll depletion despite continued biomass accumulation (Li et al., 2008) and reported a doubling of biomass after nitrogen depletion in the media, which is similar to the results obtained in this study.

4-C12 and 14-F2 show a clear increase in the percentages of specific fatty acid chains lengths, likely due to nitrogen limitation (Figure 4), an essential attribute for successful biofuel production. There is clearly a distinct FAME profile when the strains are grown in the indoor flat panel photobioreactors, which were used to generate the starting seed culture (*t* = 0 in Figure 4) as this initial profile is not recapitulated in the outdoor raceway ponds, likely due to variations in pond temperature and light intensity in comparison to the photobioreactors. From days 0 to 7 there is a marked increase in unsaturated lipids, as C16:2 appears to be replaced by C16:3 or C16:4 depending on the strain; simultaneously C18:2 is replaced by C18:3. This observation of an increase in unsaturation at cold temperatures is well documented in the literature for a variety of organisms (Neidleman, 1987; Upchurch, 2008; Sheng et al., 2011).

Although carbohydrates are not as easily utilized for biofuels relative to lipids, they represent an important fraction of biomass that can be fermented to fuels and/or higher value products. The dramatic changes in glucose content is likely due to the utilization of glucose for cell division, maintenance, and coping with the relatively cold temperatures from days 0 to 7 (Figure 3). This is supported by the increased glucose percentages after day 7, likely due to the accumulation of carbohydrates for energy storage. Galactose and mannose may contribute to the cell wall composition as suggested by Blumreisinger et al. and Takeda (Blumreisinger et al., 1983; Takeda, 1991); the portions of rhamanose (14-F2) and ribose (4-C12 and 14-F2) likely represent other structural components of the cell, or for the case of ribose, Calvin-Benson cycle intermediates and nucleic acids. A mannose polysaccharide may be used as an energy store and cell wall constituent in strain 46B-D3, given the comparatively high mannose percentage (Northcote et al., 1958). Determining the exact nature of the mannose quantified will be addressed in future research as non-starch based polysaccharides have a variety of industrial uses (Brummer et al., 2003; Abd Alla et al., 2012; Barati and Liang, 2014).

As noted above, relatively high storage carbon percentages were observed in seed cultures used to inoculate the raceway ponds, likely representing an early stationary phase culture. Inoculation of cells with relatively high stored energy reserves could prove useful as the cells will have the energy needed to quickly adapt to the new conditions present in the pond. The physiological status (growth phase and biomass composition, i.e., degree of lipid unsaturation) of seed cultures represents an area of future research optimization as cells that experience a reduced initial transfer shock as the algae are transitioned from a photobioreactor to a pond, will likely have increased productivities.

The winter productivity data reported here are unique in that metrics for biomass, along with the associated lipid and carbohydrate composition are reported under real world deployment conditions. Furthermore, algal ponds were used for growth, which is significant as techno-economic analysis has suggested that for algal biofuels to be cost competitive with traditional petroleum sources, algal ponds should be employed due to the low capital expenditure relative to photobioreactors (Davis et al., 2011; Quinn and Davis, 2015). Additionally, there is an economic benefit in using low-cost saline media, as well as positive attributes associated with the sustainability of saline water when compared to freshwater resources. The data herein provide a rare data set of algal composition and productivity during the winter growth season that researchers can utilize in techno-economic analysis.

Our data demonstrate that algal strain screening under simulated outdoor environmental conditions enables identification of strains with robust biomass productivity and biofuel precursor composition. This can be especially useful in future efforts as algae could be cultivated in a variety of geographic locations representing unique temperature and light regimes, along with other environmental variables. Importantly, we have identified a series of halotolerant strains with the highest productivities reported to date for algal winter strains grown in saline media in open raceway ponds, with biomass compositions useful for biofuels and bioproducts. These strains represent promising candidates for further development and deployment. Future studies will focus on optimization of culture conditions, down-selection of superior summer strains, and generation of facile genetic toolkits for these promising halotolerant algae.

## Data Availability

The raw data supporting the conclusions of this manuscript will be made available by the authors, without undue reservation, to any qualified researcher.

## Author Contributions

LD, MP, and MG conducted experimental design and wrote the manuscript. LD performed strain screening, characterization, and down-selection. SVW directed FAME and carbohydrate analysis. HG and JM directed the outdoor deployment. All authors reviewed and revised the manuscript.

## Conflict of Interest Statement

The authors declare that the research was conducted in the absence of any commercial or financial relationships that could be construed as a potential conflict of interest.

## Supplementary Material

FIGURE S1 Photobioreactor schematic for mid-throughput, simulated outdoor screening of algal cultivars.

TABLE S1 Algal down-selection criteria.
