# Supplementary figures and images for: Down-Selection and Outdoor Evaluation of Novel, Halotolerant Algal Strains for Winter Cultivation
Source: Front Plant Sci. 2018 Oct 29;9:1513. doi: 10.3389/fpls.2018.01513 (PMC6232915; doi:10.3389/fpls.2018.01513)

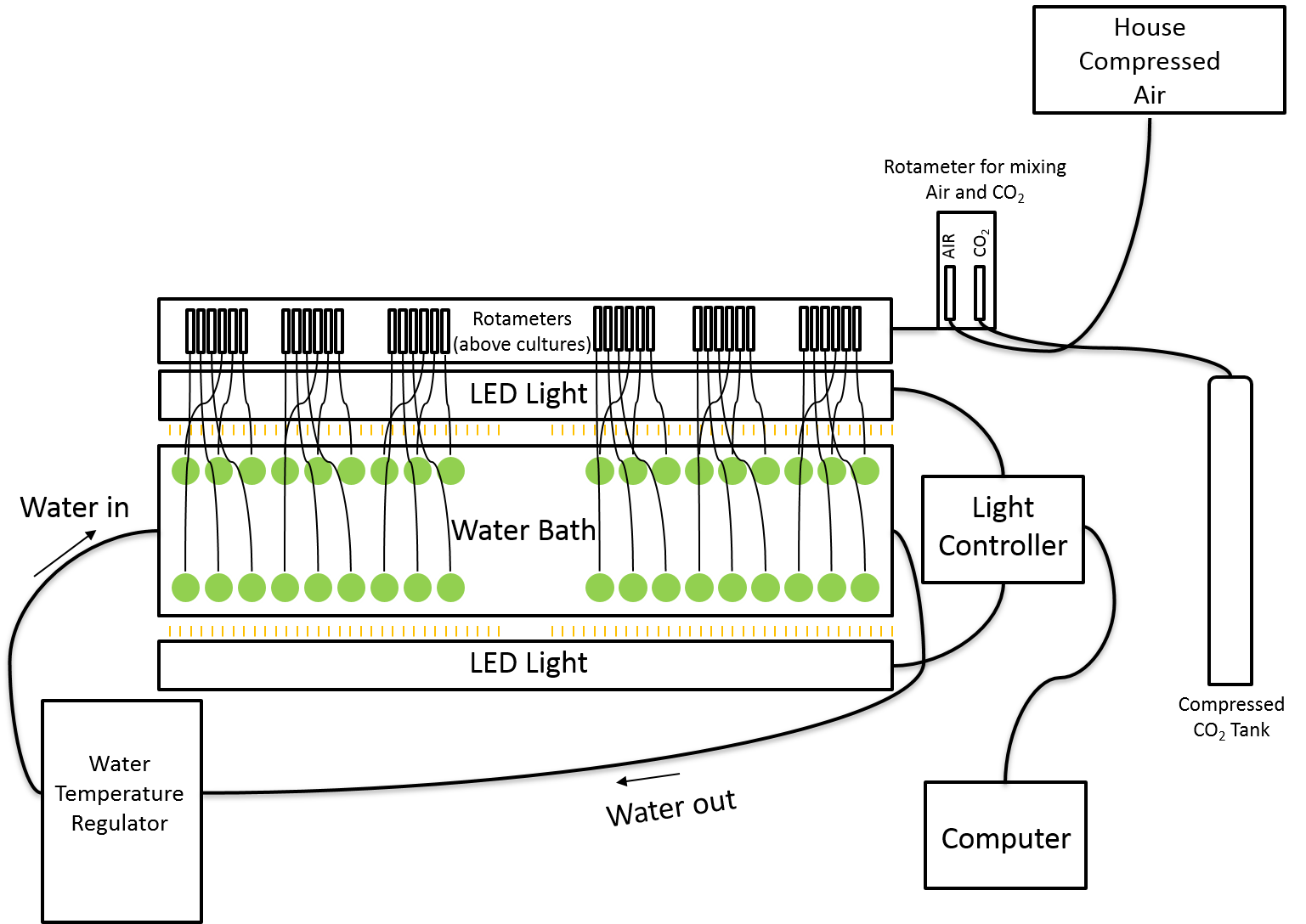

Supplement: FIGURE S1 — Photobioreactor schematic for mid-throughput, simulated outdoor screening of algal cultivars. [file Image_1.PNG]
